# The feasibility, safety, and efficacy of upper limb garment-integrated blood flow restriction training in healthy adults

**DOI:** 10.1186/s40814-022-00995-4

**Published:** 2022-02-08

**Authors:** Bhavit Dhokia, Elspeth Olivia Mabin, Warren Jeremy Bradley, Bradley Stephen Neal

**Affiliations:** 1 Sports and Exercise Medicine, School of Medicine and Dentistry, William Harvey Research Institute, Queen Mary University of London, London, UK; 2 Hytro Ltd., 2 Park Court, Pyrford Road, West Byfleet, Surrey UK; 3 School of Sport, Rehabilitation and Exercise Sciences, University of Essex, Wivenhoe Park, Colchester, UK

**Keywords:** Safety, Feasibility, Blood flow restriction, Kaatsu training, Occlusion training, BFR-integrated

## Abstract

**Background:**

Blood flow restriction training (BFR) has been demonstrated to increase muscle hypertrophy and strength, but has logistical and cost barriers. Garment-integrated BFR has the potential to reduce these barriers by lowering equipment demands and cost. The primary aim of the study was to explore the feasibility of garment-integrated BFR in the upper limb of healthy adults, with a secondary aim of exploring safety and efficacy.

**Methods:**

Physically active and otherwise healthy participants with no previous experience with BFR were sought. Eligible participants completed a five-week garment-integrated BFR programme that involved completing two sessions per week. Feasibility was determined by a priori defined thresholds for recruitment, adherence to the garment-integrated BFR programme, and data collection. Safety was determined by recording adverse events and by monitoring for total arterial occlusion pressure using a fingertip pulse oximeter. Efficacy was determined by measuring push-ups to volitional failure, arm girth, and number of prescribed repetitions completed. Feasibility and safety outcomes were reported descriptively or as a proportion with associated 95% confidence intervals (95% CI). Mean change, 95% CIs, and associated effect sizes were calculated for efficacy outcomes.

**Results:**

Twenty-eight participants were included (15 men, 13 women; mean age 31.6 years [±9.1]) and 27 successfully completed the study. Participants were successfully recruited within three months and 278/280 sessions were successfully completed (adherence=99.3%, 95% CI 97.4%, 99.9%). Minimal adverse events were reported; one incident of localised bruising (0.36%, 95% CI 0.06%, 2.0%) and three incidences of excessive pain during or post-exercise from two separate participants (1.07%, 95% CI 0.03%, 3.1%). 82/2240 pulse oximeter readings were not recorded (3.7%, 95% CI 2.9%, 4.5%). Mean push-ups to volitional failure increased by 40% (mean change=8.0, 95% CI 6, 10, *d=1.40*). Mean arm girth and number of prescribed repetitions completed were unchanged.

**Conclusions:**

Garment-integrated BFR is feasible and has no signal of important harm in the upper limb of healthy adults, and could proceed to a future trial with stop/go criteria for randomisation. Further work is required to investigate the efficacy of garment-integrated BFR and determine its equivalence or superiority compared to existing BFR methods.

## Key messages regarding feasibility

- It was uncertain if participants would be adherent to garment-integrated blood flow restriction training (BFR) or whether it could be safely applied.
- Garment-integrated BFR was found to be feasible, with participants adherent to its use, and there was no signal of important harm.
- Garment-integrated BFR can now be feasibly applied in a randomised controlled trial design to determine efficacy when compared to either a wait and see control, or another form of BFR.

## Background

Blood flow restriction (BFR) training originated in Japan and is known as “kaatsu” training, which translates as “added pressure” [1]. BFR involves the partial occlusion of limb vasculature using a torniquet or cuff, positioned at the most proximal part of the limb being trained [2]. Current application of BFR involves either the use of a pneumatic cuff or a simple tourniquet (e.g., rubber tubing). Pneumatic cuffs allow for standardisation of occlusion pressure and limb placement, but are expensive (£350–£10,000) and require specialist equipment and supervision that limits their wider application. Simple tourniquets can be unsafe as they do not allow for standardisation of limb placement or the reproducible determination of occlusion pressure [3].

When BFR is used by trained professionals it is typically with specialist equipment to accurately regulate the applied occlusion pressure. Most research to date uses a defined percentage of total arterial occlusion pressure, which is not a realistic expectation of the practical application of BFR outside of a laboratory setting [4]. Comparable muscular responses in strength and hypertrophy are reported at varying levels of occlusion pressure [5, 6], questioning the need for defined measurement of a percentage of total arterial occlusion pressure, beyond ensuring sub-occlusive pressure for safety purposes [6].

Garment-integrated BFR has been developed to provide an inexpensive and safe method of BFR application. This allows for consistent placement of an integrated strap of a standardised width, and the subjective but reproducible determination of sub-occlusive pressure [7]. Garment-integrated BFR can also be used without the supervision of a qualified professional, increasing the potential application in a variety of settings. The safe application of BFR has been reported in many different populations with a paucity of minor (muscle soreness) and serious (rhabdomyolysis, thrombosis) side-effects [1, 8]. It is yet to be determined if people would be adherent to garment-integrated BFR, or what adverse events may arise from its use.

Low-load resistance exercise (~30% one-repetition maximum) facilitates muscle endurance rather than strength [9], but when combined with BFR, promotes greater strength adaptations and hypertrophy than low-load resistance exercise in isolation [10, 11]. Low-load resistance exercise combined with BFR can also facilitate comparable levels of hypertrophy to high-load resistance exercise (~70% one-repetition maximum; [10]). Many individuals are unable to perform high-load resistance exercise for a variety of logistical and safety reasons. BFR has previously been reported to facilitate hypertrophy and improve functional capacity in both healthy [11] and injured [12] populations, but this is yet to be explored using garment-integrated BFR.

The primary aim of this study was therefore to explore the feasibility of garment-integrated BFR in the upper limb of healthy adults, to inform upon future larger scale studies. A secondary aim was to explore the safety and efficacy of garment-integrated BFR. The null hypothesis was that garment-integrated BFR of the upper limb would be infeasible in healthy adults.

## Methods

An observational feasibility and safety cohort study was conducted.

### Ethical approval

Ethical approval was granted by the Queen Mary Ethics of Research Committee (QMREC2018/48/054).

### Participants

Participants were recruited as a sample of convenience and provided written informed consent prior to study commencement using Google Forms (Google LLC, California, USA). The required sample size was based on previous guidelines for feasibility studies [13], with a minimum of 12 male and 12 female participants sought. Participants were eligible for inclusion if they were over the age of 18 and currently in good health, injury-free in their upper limbs, and willing to perform two BFR sessions per week and cease other forms of upper limb exercise for the study duration. Participants were excluded if they had any history of deep vein thrombosis (DVT) or pulmonary embolism (PE), a previous diagnosis of rhabdomyolysis, haemorrhagic or thrombotic stroke, previous surgery in the past six weeks, were currently or recently pregnant, or had a family history of any blood clotting disorder. Participants were also excluded if they had any prior experience with BFR.

#### Demographics

Eligible participants self-reported their age (in years), height (to the nearest cm), mass (to the nearest kg), and activity level using the Tegner scale. Combining both work and sports activities, the Tegner scale is a reliable and valid measure to reflect the average activity levels of recruited participants [14].

### Experimental protocol

**Fig. 1:**
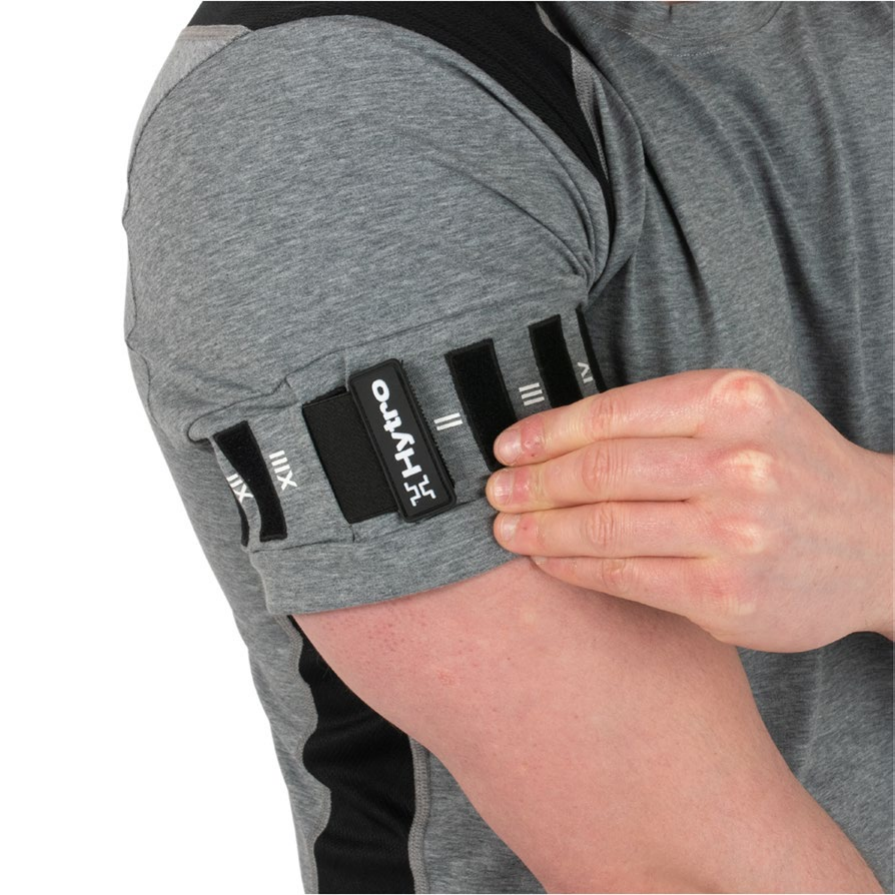
Garment-integrated BFR with Velcro mechanism

The study was conducted online using Microsoft Teams (1.400.11161, Microsoft, Washington, USA) due to the SARS-CoV-2 pandemic. All included participants followed a five-week upper limb BFR programme consisting of two sessions per week and a total of ten sessions, and were advised to avoid other forms of upper limb exercise for the duration of the study. Participants were provided with a garment with integrated BFR (Hytro Limited, London, UK). This garment uses a standardised elastane strap (width 4cm) located at the most proximal part of the upper limb and secured with a Velcro mechanism (YKK Fastening Corp, Tokyo, Japan; see Fig. 1) to allow for standardisation of compression stimulus. Participants were also provided with a standardised resistance band (male=red [tension 7–16kg] and female=yellow [tension 2–9kg]), a flexible tape measure, and a fingertip pulse oximeter (model number LK88, ViATOM, China). Participants had an initial familiarisation meeting with a researcher (BD or EM), where they were introduced to the BFR programme and protocol. Participants were then instructed to pull the BFR strap on their dominant arm to its maximal position (reflecting 100% compression stimulus and 10/10 on a numerical rating scale), before releasing to a perceived 50% compression stimulus (5/10 on a numerical rating scale), noting the corresponding number on the Velcro mechanism. Participants then underwent a three-minute passive BFR session with their arms stationary at 50% compression stimulus to familiarise them to the sensation of BFR, before releasing. Participants finally completed four sets of a single exercise (banded bicep curls) at 50% compression stimulus, resting for 30-s between sets. Participants were instructed to determine the presence of a pulse using a fingertip pulse oximeter immediately after applying their BFR strap (but before starting to exercise) and immediately after the exercise was completed (but before releasing their BFR strap) for familiarisation.

### BFR programme

Each BFR session involved four exercises (push-ups, banded bent over rows, banded triceps extensions, and banded bicep curls). Male participants were instructed to complete full push-ups, whilst female participants were instructed to complete kneeling push-ups. Adhering to the protocol described by Patterson et al., [1], participants were required to complete four sets of each exercise (30/15/15/15 repetitions), with a 30-s rest interval between sets and a two-min rest interval between exercises. The two-minute rest interval between exercises was designed to allow for limb reperfusion [1]. If participants reached volitional failure prior to the prescribed number of repetitions in a set, they were instructed to record their number of successful repetitions. Participants were instructed to tighten their BFR strap to a perceived compression stimulus of 50% for their first session (5/10 on a numerical rating scale) and increase to 60% for their second session (6/10 on a numerical rating scale) as a familiarisation week. Participants were then instructed to increase to a perceived compression stimulus of 70% (7/10 on a numerical rating scale) for the remaining eight sessions (weeks 2–5). The BFR strap was to be applied prior to commencing an exercise and remain secured for all four sets (i.e., 75 repetitions), before releasing at the start of the two-min rest interval between exercises.

### Feasibility outcomes

Successful recruitment was determined by the time within which a minimum of 24 participants could be recruited, with a maximum of three months defined a priori.

Successful adherence was determined by monitoring the number of sessions completed by each participant (x/10), with a minimum of 80% required a priori. Successful data collection was determined by outcome measure capture, with a minimum of 80% required a priori.

### Safety outcomes

Safety was determined by recording the prevalence of adverse events during and after BFR training and by monitoring for potential total arterial occlusive limb pressure reflected by an absent pulse using a fingertip pulse oximeter.

#### Questionnaire

Potential adverse events that could reflect thrombosis, ischaemia, or rhabdomyolysis [15] were monitored (Table 1), with participants instructed to record and report any response that was atypical for them post-exercise. Participants completed a safety questionnaire after each BFR session and attended a weekly virtual meeting with a researcher (BD/EM) to report any adverse events that occurred during the preceding week. Participants were also instructed to report anything of immediate concern to the primary investigator (BSN) via email or telephone.

**Table 1:** Adverse events monitored for the duration of the BFR programme

Adverse events during exercise	Adverse events post exercise
Excessive pain (subjective severity)	Excessive pain (subjective severity)
Chafing/abrasions	Shortness of breath
Bruising/pressure marks	Whole arm swelling
	Chafing/abrasions
	Bruising/pressure marks
	Persistent tingling/paraesthesia
	Numbness/loss of sensation

#### Pulse oximetry

Participants were required to confirm the presence of an upper limb pulse by taking a fingertip pulse oximeter reading before commencing each exercise (once their BFR strap had been applied at the required perceived compression) and once each exercise had been completed (immediately prior to releasing their BFR strap and commencing their rest period). The presence of a pulse, determined using a fingertip pulse oximeter, has been reported to be a valid method of ensuring sub-occlusive arterial pressure in the upper limb when compared to the gold standard of ultrasound doppler [16].

### Efficacy outcomes

#### Push-ups to volitional failure

All participants performed a single push-up to volitional failure test in their familiarisation meeting, but before their BFR familiarisation. Total push-ups were observed and recorded by the researcher during the video call, and the test was ceased once participants were unable to complete a full repetition meeting the minimum movement standard of 90° elbow flexion (i.e., volitional failure). The speed that participants performed their push-ups at was not controlled for, and no BFR compression was applied during the test. This test was then repeated in the final meeting after the five-week BFR programme, within one week of the participant’s final BFR session. Push-ups to volitional failure was chosen as a proxy measure of strength as it has been reported to correlate well with a one repetition maximum bench press using an equivalent load [17]. It could also be performed virtually and without requiring participants to attend a human performance laboratory during the SARS-CoV-2 pandemic.

#### Arm girth

Arm girth was measured by the participant using a flexible tape measure according to the International Society for the Advancement of Kinanthropometry (ISAK) guidelines [18]. Participants were instructed to measure from their acromion process to their cubital fossa on their right arm, marking the midpoint. Participants were then instructed to take a circumferential measurement of their arm at this point to the nearest 0.5cm, with their arm relaxed in the anatomical position [18].

#### Number of prescribed repetitions completed

The total number of repetitions completed were compared from week two to week five, excluding week one as a familiarisation week, as a measure of muscular endurance [19].

### Statistical analysis

Data were collected and collated using a customised spreadsheet (Microsoft Excel 16.0.13426320270, Microsoft, Washington, USA). Feasibility and safety data were analysed using Microsoft Excel. Safety outcomes were calculated by dividing the incidence of reported adverse events by the total number of BFR sessions (x/280) and expressed as a percentage. Adherence outcomes were calculated by dividing the number of completed sessions by the total number of prescribed sessions (x/280) and expressed as a percentage. 95% confidence intervals (95% CI) were also calculated for all feasibility and safety outcomes.

Efficacy data were analysed using JAMOVI (v.1.6.23, the JAMOVI project, Sydney, Australia). Mean change and associated standard deviation (SD) were calculated for push-ups to volitional failure and arm girth (follow-up—baseline), and total number of repetitions completed (week five–week two). A Shapiro-wilk normality test was conducted to determine if data were normally distributed. As a feasibility study not powered a priori to detect statistical significance, dependent samples *t*-tests were not performed, and *p*-values not reported, because of the potential for type II error and to avoid giving the impression of there being robust findings from a feasibility design. Instead, mean change with 95% CIs and effect sizes [20] were calculated. If normally distributed, a Cohen’s *d* was calculated and interpreted as trivial (<0.2), small (0.2–0.49), medium (0.5–0.79), and large (≥0.8; [21]) and if non non-normally distributed, a rank biserial correlation (RBC) was calculated and interpreted as strong positive correlation (1.0), no correlation (0), and strong negative correlation (−1.0; [22]).

## Results

### Participants

Twenty-eight participants (15 males, 13 females) were recruited and 27 (14 males, 13 females) completed the study and are included in all analyses. One male participant failed to appropriately follow the protocol and did not complete the study. Cohort demographic data are presented in Table 2.

**Table 2:** Baseline demographic data

	Age (years)		Height (cm)		Mass (kg)		Tegner scale	
	Male	Female	Male	Female	Male	Female	Male	Female
**Mean ± SD**	31.1 ± 7.4	32.1 ± 10.9	180.4 ± 6.9	166.7 ± 4.8	79.4 ± 10.4	63.9 ± 6.1	6.9 ± 0.6	6.2 ± 0.7

### Feasibility outcomes

Twenty-eight participants were successfully recruited and enrolled within 3 months, commencing on March 08, 2021, and ceasing on March 25, 2021.

278/280 sessions were successfully completed (adherence=99.3%, 95% CI 97.4%, 99.9%). One male participant did not complete the final week of the protocol.

Full data were collected from 27/28 participants (96.4%, 95% CI 82.3%, 99.4%).

### Safety outcomes

#### Adverse events

One participant reported a single incidence of excessive pain during exercise (0.36%, 95% CI 0.06%, 2.0%). Two participants reported single incidences of excessive pain post-exercise (0.72%, 95% CI 0.07%, 2.6%). One participant reported a single incidence of bruising in the locality of the BFR strap post-exercise (0.36%, 95% CI 0.06%, 2.0%). No other adverse events were reported.

#### Pulse oximetry

An absent pulse oximeter reading was reported by four participants either before or after an exercise a total of 82 (out of 2240) times (3.7%, 95% CI 2.9%, 4.5%), with one participant reporting an absent pulse oximeter reading 56 times and another participant reporting an absent pulse oximeter reading on 24 occasions. The remaining two incidences of absent pulse oximeter readings came from two separate participants.

### Efficacy outcomes

#### Push-ups to volitional failure

**Fig. 2:**
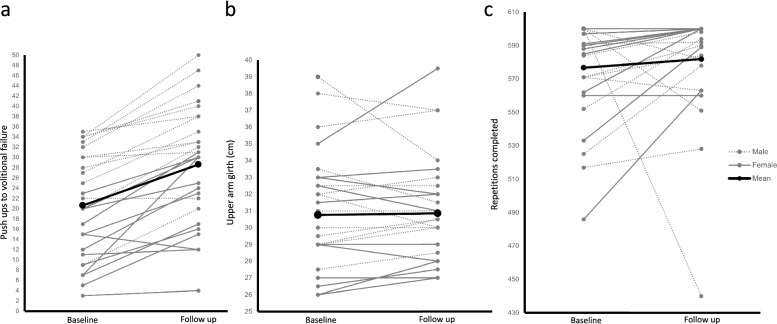
**a**–**c** Individual participant data at baseline and follow-up for push-ups to volitional failure and arm girth, and from weeks two to five for total repititions completed

Shapiro-Wilk test for normality indicated normally distributed data (*p*=0.7). The mean number of push-ups increased from baseline (21 ± 10) to follow-up (29 ± 11), reflecting a mean increase of 40% and a mean change of 8.0 (95% CI 6, 10, Cohen’s *d* 1.40; see Fig. 2a and Table 3).

**Table 3:** Mean change, 95% CIs, and effect sizes for efficacy data

	Mean change	Lower 95% CI	Upper 95% CI	Effect size	
**Push-ups to volitional failure**	8.0	6.0	10.0	Cohen’s *d*	1.40
**Arm girth**	<0.001	−0.50	1.00	Rank biserial correlation	0.08
**Total repetitions**	11.50	0.50	28.00	Rank biserial correlation	0.50

#### Arm girth

Shapiro-Wilk test for normality indicated non-normally distributed data (*p*=0.01). No change in arm girth was observed from baseline (30.8 ± 3.6) to follow-up (30.9 ± 3.3), reflecting a mean change <0.001 (95% CI −0.5, 1.0, RBC 0.08; see Fig. 2b and Table 3).

#### Number of prescribed repetitions completed

Shapiro-Wilk normality test indicated non-normally distributed data (*p*≤0.001). The total number of repetitions completed each week increased from week two (576.7 ±30.5) to week five (581.9 ± 34.0), reflecting a mean change of 11.5 (95% CI 0.50, 28.00, RBC 0.50; see Fig. 2c and Table 3).

## Discussion

This study aimed to explore the feasibility, safety, and efficacy of garment-integrated BFR in the upper limb of healthy adults. Consistent with our hypotheses, garment-integrated BFR was identified to be feasible, with no signal of important harm. Secondary increases were observed in push-ups to volitional failure and total repetitions completed, but not participant-measured arm girth. 

### Feasibility

All three of the a priori defined feasibility outcomes were satisfied. The minimum number of required participants were comfortably recruited within three months, and complete data were obtained from 96% of participants. Adherence to the garment-integrated BFR protocol used was high (99.3%), which is comparable to adherence rates reported by other BFR feasibility studies in clinical populations [23]. The one male participant who failed to complete the final two BFR sessions wished to return to his typical upper limb training routine, which led to his withdrawal. Overall, this gives a high degree of confidence that garment-integrated BFR could be scaled up and investigated using a randomised controlled trial design.

### Safety

No signal of important harm was identified when garment-integrated BFR was applied to the upper limb of healthy adults, reflected by the minimal presence of adverse events and confirmation of sub-occlusive pressure. Muscle soreness is a common side effect of low load resistance exercise combined with BFR [1], which may persist for up to 72 h [15]. Two participants (8%) in this study reported excessive muscle pain post-exercise. Upon further exploration, one of these participants failed to cease the protocol at the point of volitional failure and instead completed the maximum prescribed repetitions with additional unprescribed rest intervals. The other participant went against the advised study protocol and combined his BFR training with a sudden return to regular golf as the UK SARS-CoV-2 pandemic rules were eased (36 holes in a five-day period). After a week of prescribed rest and reinforcement of instructions, these participants returned to the protocol without any further excessive muscle soreness, indicating that their initial episodes are unlikely to be the direct result of garment-integrated BFR.

Whilst there is little consistency amongst the literature regarding optimal BFR occlusion pressure [6], a maximum of 80% arterial occlusive pressure is advocated when combining BFR with resistance exercise. An occlusive pressure greater than this is not advised by the most recent BFR position statement to minimise the potential for more serious adverse events [1]. In the absence of a gold standard of ultrasound doppler, pulse oximetry was used to ensure sub-occlusive arterial pressure [16]. A successful pulse oximeter reading was achieved 2158/2240 times, with a pulse oximeter reading absent only 82 times (3.7%). Upon further exploration, one participant accounted for 56/82 absent readings, which was rectified by the provision of a new pulse-oximeter, suggesting equipment failure. A further participant accounted for a further 24 absent pulse oximeter readings, likely to be explained by their use of acrylic false nails [24]. Pulse oximetry and participant determined compression stimulus is a method with no signal of important harm that can be used in future trials of garment-integrated BFR to ensure sub-occlusive pressure, and the secure Velcro mechanism allows for consistent replication of the required compression stimulus.

### Efficacy

Whilst not designed for hypothesis testing, a mean increase of eight push-ups to volitional failure was observed. This may reflect an improvement in muscle strength, as push-ups are a valid predictor of upper body strength [17]. An increase in strength would be expected after combining low load resistance training with BFR [10]. The absence of a control group in this study means that it is impossible to separate a dependent training effect from an independent effect of BFR. With feasibility established, future research should look to investigate the efficacy of garment-integrated BFR in an adequately powered trial with an appropriate control.

No change in participant-measured arm girth was observed amongst the participants in this study. Because of the limitations placed on clinical research during the SARS-CoV-2 pandemic, participants were required to measure their own arm girth using a flexible tape measure whilst observed by a researcher, leading to questionable reliability. Limb girth is also affected by several variables beyond muscle hypertrophy, including water retention and adipose tissue. Future studies are advised to apply valid and reliable methods of body composition testing, such as muscle cross-sectional area magnetic resonance imaging [25, 26] once the pandemic-related restrictions on clinical research are lifted.

A modest mean increase of 11.5 repetitions was observed between weeks two and five. The total number of repetitions completed is a valid predictor of muscular endurance [19], and an improvement in muscular endurance would be expected when combining low load resistance training with BFR [10]. The ceiling of the prescribed repetitions was 600 repetitions per week, and eight participants were completing this maximum volume from week two, indicating that the prescribed training protocol was not an appropriate challenge for almost one third of the cohort. Participants were unable to access gym facilities and perform a more specific protocol reflective of their baseline condition due the SARS-CoV-2 pandemic. Future studies should look to explore low load resistance training combined with BFR that is specific to the individual, and include a muscular endurance test using a fixed percentage of one repetition maximum to volitional failure [27], to appropriately evaluate the effect of garment-integrated BFR on muscular endurance.

### Interpretation

Recruitment, adherence, and data collection were all successful, meaning that a future larger scale trial is feasible. The feasibility of randomisation was not investigated in this study, and a future trial should therefore have appropriate stop/go criteria should initial randomisation prove infeasible. Garment-integrated BFR demonstrated no signal of important harm in the upper limb of healthy adults when a pulse oximeter and subjective measure of compression stimulus is used. It is plausible that this safety outcome is generalisable to wider populations beyond young and otherwise healthy adults, but future trials should look to confirm this ahead of commencement if investigating a different population (e.g., older adults).

## Conclusions

Garment-integrated BFR is a feasible approach in the upper limb of healthy adults and could proceed to a future trial with stop/go criteria for randomisation. Garment-integrated BFR can be applied in a resistance training setting using existing BFR protocols, and there was no signal of important harm in the investigated young and otherwise healthy cohort. Further work is required to investigate the efficacy of garment-integrated BFR and determine its equivalence or superiority compared to existing BFR methods with respect to facilitating muscle hypertrophy, strength, and endurance.

## Data Availability

Raw data can be provided upon request.
